# Towards clinical implementation of automated segmentation of vestibular schwannomas: a reliability study comparing AI and human performance

**DOI:** 10.1007/s00234-025-03611-3

**Published:** 2025-04-04

**Authors:** Stefan Cornelissen, Sammy M. Schouten, Patrick P. J. H. Langenhuizen, Henricus P. M. Kunst, Jeroen B. Verheul, Peter H. N. De With

**Affiliations:** 1 Gamma Knife Center, Department of Neurosurgery, Elisabeth-TweeSteden Hospital, Tilburg, Netherlands; 2 Department of Electrical Engineering, Eindhoven University of Technology, Eindhoven, Netherlands; 3 Department of Otolaryngology, Radboud University Medical Center, Nijmegen, Netherlands; 4 Department of Otolaryngology, Maastricht University Medical Centre+, Maastricht, Netherlands

**Keywords:** Vestibular schwannoma, Automatic segmentation, Clinimetric reliability, Inter-observer variability, MRI

## Abstract

**Purpose:**

To evaluate the clinimetric reliability of automated vestibular schwannoma (VS) segmentations by a comparison with human inter-observer variability on T1-weighted contrast-enhanced MRI scans.

**Methods:**

This retrospective study employed MR images, including follow-up, from 1,015 patients (median age: 59, 511 men), resulting in 1,856 unique scans. Two nnU-Net models were trained using fivefold cross-validation to create a single-center segmentation model, along with a multi-center model using additional publicly available data. Geometric-based segmentation metrics (e.g. the Dice score) were used to evaluate model performance. To quantitatively assess the clinimetric reliability of the models, automated tumor volumes from a separate test set were compared to human inter-observer variability using the limits of agreement with the mean (LOAM) procedure. Additionally, new agreement limits that include automated annotations are calculated.

**Results:**

Both models performed comparable to current state-of-the-art VS segmentation models, with median Dice scores of 91.6% and 91.9% for the single and multi-center models, respectively. There is a stark difference in clinimetric performance between both models: automated tumor volumes of the multi-center model fell within human agreement limits in 73% of the cases, compared to 44% for the single-center model. Newly calculated agreement limits including the single-center model, resulted in very high and wide limits. For the multi-center model, the new agreement limits were comparable to human inter-observer variability.

**Conclusion:**

Models with excellent geometric-based metrics do not necessarily imply high clinimetric reliability, demonstrating the need to clinimetrically evaluate models as part of the clinical implementation process. The multi-center model displayed high reliability, warranting its possible future use in clinical practice. However, caution should be exercised when employing the model for small tumors, as the reliability was found to be volume-dependent.

## Introduction

Vestibular schwannomas (VS) are uncommon benign intracranial tumors emerging from Schwann cells of the vestibulocochlear nerve [1]. In the last decades, tumor management has shifted from microsurgery towards more conservative approaches [2]. Tumor observation (or wait-and-scan) is increasingly employed for patients with small tumors, since symptoms typically do not improve after treatment [2, 3]. When these (smaller) tumors do progress, stereotactic radiosurgery may be utilized to stabilize such tumors [1, 4]. Consequently, reliable tumor monitoring is essential for clinical decision making, since it is important to differentiate between stable and growing tumors in patients who are either observed or have been treated radiosurgically.

Tumor monitoring is commonly performed by linear measurement in clinical practice, as volumetric analyses are considered to be too time-consuming [5–7]. However, studies have shown that linear measurements are substantially less sensitive in detecting tumor progression, in comparison to volumetric measurements [5–9]. Therefore, with the recent surge in research in artificial intelligence (AI), studies have been conducted on the use of AI to alleviate these problems in VS monitoring, by investigating automated volumetric annotation algorithms [10–14]. The developed algorithms demonstrate a high performance in terms of commonly used segmentation performance metrics, like the Dice score and average symmetric surface distance (ASSD). As such, these results indicate the high potential of using such algorithms in a clinical setting. This performance score and its potential are further substantiated by two thorough systematic technical reviews on automatic VS annotation [15, 16].

However, in clinical routine practice it is primarily important to detect changes in volume over time. This criterion is not reflected by the commonly used segmentation performance metrics, such as Dice and ASSD. These metrics provide information on the delineation accuracy in terms of their geometry, but lack the capability to differentiate random errors from the inherent inter-observer variability in human annotations [17–19]. Additionally, depending on the clinical domain interest, these geometric-based metrics may not reflect the requirements for clinical practice and thereby may lack sufficient clinical relevance [18, 20–23]. In the case of VS, the commonly used segmentation metrics only reflect the geometric similarity of the automatic annotations, whereas for clinical routine practice the quality of tumor volumetry is of much higher relevance. This notion is disregarded in recent literature reviews [15, 16], but is addressed in work by Neve et al. using qualitative methods [13].

An essential step towards integrating these automated volumetric annotation algorithms in clinical practice involves therefore quantitatively establishing the clinical reliability of these tools with respect to human annotators. This can be achieved through the assessment of clinimetric reliability parameters, which is a quantitative approach to the analysis of both clinical and clinically relevant data [24]. In this study, the measurement error and smallest detectable change (or agreement limits) are of particular interest. These parameters provide minimum threshold values for change, thereby ensuring that the observed change is legitimate and not a result of measurement error [25, 26]. By employing these clinimetric parameters, it is possible to evaluate the reliability of VS annotation algorithms in clinically relevant terms. To this end, the established automatic volumetric annotation methods are exploited to investigate their clinimetric reliability. Automated annotations are subsequently compared to human agreement limits by means of an inter-observer variability study, comprising both human and algorithmic annotators.

## Materials and methods

This retrospective study was conducted at Elisabeth-TweeSteden Hospital (ETZ) in Tilburg, The Netherlands, a tertiary referral hospital for VS. Institutional review board approval was obtained and the requirement for informed consent was waived.

### Datasets

**Fig. 1:**
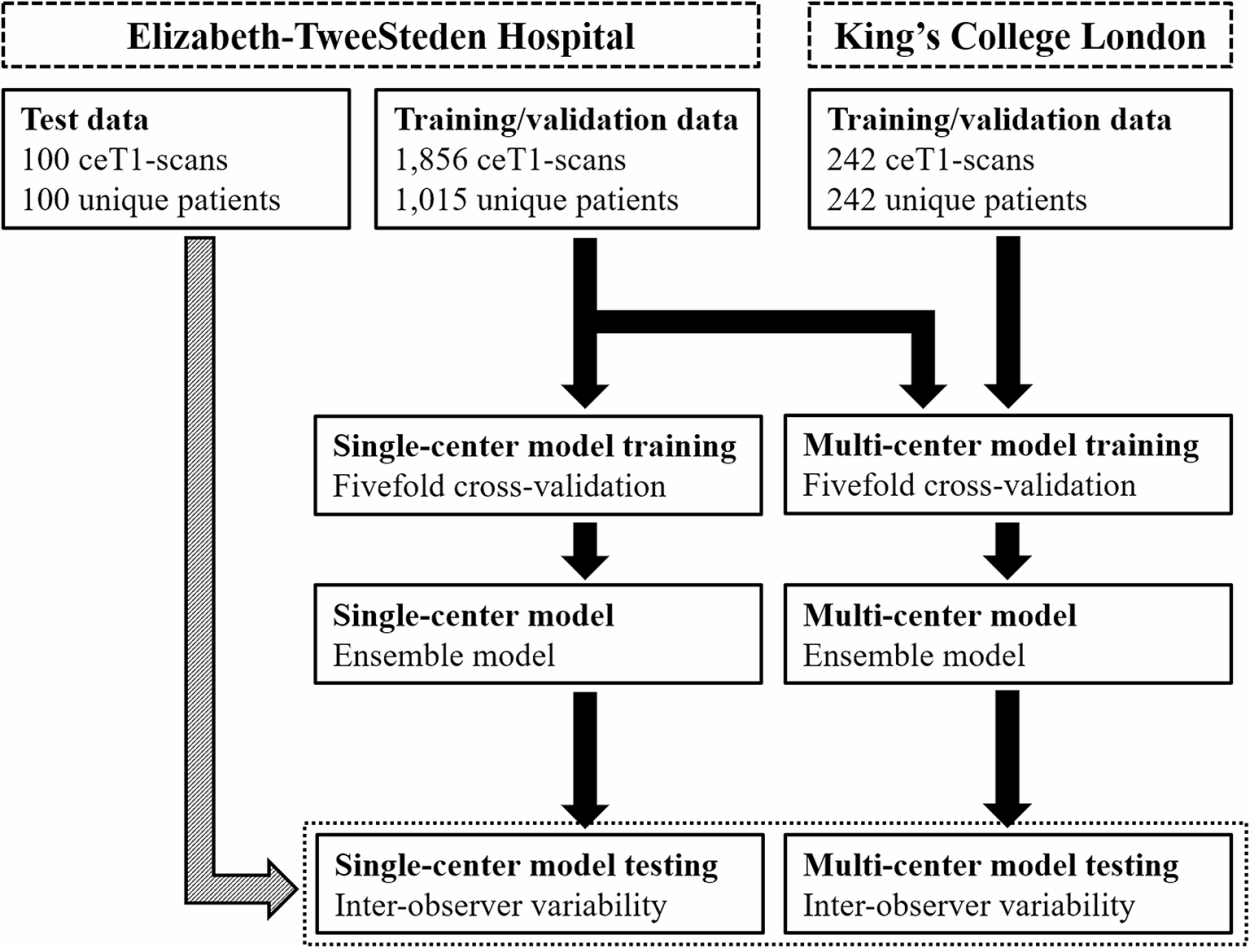
Flowchart of the employed datasets. Both models are trained using fivefold cross-validation, where multiple instances of an individual patient, due to the inclusion of both treatment and follow-up imaging, are always within the same fold. Model performance is evaluated on a separate test set from Elisabeth-TweeSteden Hospital [26]. The King's College London data refers to the open-access dataset by Shapey et al. [29]

Three different datasets were selected for our study: two as training and validation sets, and one as a test set for model evaluation and inter-observer assessment (see Fig. 1).

ETZ has established an extensive database of patients with unilateral sporadic VS treated with Gamma Knife radiosurgery (GKRS) [27, 28]. Patients underwent MRI as part of GKRS treatment planning (2002–2020) and during follow-up (2002–2020). All tumors were annotated and volumetrically analyzed using the GKRS treatment planning software (GammaPlan, Version 11, Elekta AB, Stockholm, Sweden) by the treating physician before treatment and by experienced researchers during follow-up. We employ a part of this data for our training and validation dataset, resulting in 1,856 ceT1-scans of 1,015 unique patients (median age, 59 years; 511 men).

To complement our data to a multi-center setting, the open-access dataset from King’s College London (KCL) has been used [29]. This dataset consists of 242 ceT1-scans (including annotations) of unique patients with unilateral sporadic VS undergoing GKRS treatment planning.

For model testing and inter-observer evaluation, we employ volumetric annotations of five observers in 100 VSs from ETZ, which have been previously reported [26]. Herein, each observer annotated all tumors independently without prior information (i.e. earlier annotations). There is no patient overlap between any of the datasets. Patient characteristics and imaging parameters of all three datasets are summarized in Table 1.

### Annotation models and image preprocessing

Two automated annotation models are developed for this study: a single-center model using only ETZ data, and a multi-center model using both ETZ and KCL data. For both models, nnU-Net is employed, a commonly used self-configuring framework for biomedical image segmentation using convolutional neural networks with a proven track record in automatic VS annotation [13, 14, 30, 31]. This framework automatically selects preprocessing and postprocessing steps for the data, creates the model architecture, and tunes the hyperparameters. In both cases, a three-dimensional nnU-Net model is created with six encoder and decoder layers.

**Table 1:** Patient characteristics and imaging parameters of all three selected datasets

	Training and validation datasets		Test dataset
	ETZ dataset	KCL dataset	Inter-observer dataset
Number of scans Unique patients Follow-up scans per patient^†^	1,856 1,015 1 (0–9)	242 242 N/A	100 100 N/A
Patient characteristics			
Median tumor volume [mm^3^] ^*^ Median age at treatment Sex [M/F]	1,530 (654-3,643) 59 (51–67)^*^ 511/504	1,360 (630-3,170) 56 (24–84)^†^ 95/147	903 (193-3,101) 58 (50–67)^*^ 59/41
Imaging characteristics^†^			
Echo time [msec] Repetition time [msec] Field strength [T] Slice thickness [mm] Voxel spacing [mm]	4.67 (1.82–250.0) 25.0 (8.18-5,556.6) 1.5 (1.0–3.0) 1.5 (0.19–6.6) 0.82 (0.34-1.0)	2.97 (-) 1,900.0 (-) 1.5 (-) N/A (1.0-1.5) 0.4 (-)	4.60 (3.9–6.9) 25.0 (8.4–26.6) 1.5 (1.0–3.0) 1.6 (0.8-2.0) 0.78 (0.25-1.0)

Note. –

^*^) Data are presented as median with inter-quartile range

^†^) Data are presented as median with range

ETZ = Elisabeth-TweeSteden Hospital, KCL = King’s College London

As part of the image preprocessing, images are resampled using third-order spline interpolation to the median voxel spacing of the dataset. Z-score normalization is employed on each image separately to mitigate MRI signal-intensity variations among different scanners and imaging protocols.

Fivefold cross-validation is used for model training, resulting in an ensemble model. Multiple instances of an individual patient, due to the inclusion of both treatment and follow-up imaging, are always within the same fold. During training, images are randomly cropped as a result of hardware limitations. In both cases, a batch size of two is used. The models are trained from scratch with He initialization for 1,000 epochs and optimized using stochastic gradient descent with the loss function defined as the sum of the Dice loss and the cross-entropy loss. Training is performed on a Quadro RTX 6000 GPU (Nvidia Corp., Santa Clara, CA, USA) with 24-GB VRAM using the PyTorch library (Version 2.0.1) and CUDA Toolkit (Version 11.7).

### Segmentation performance evaluation

The previously obtained inter-observer data of 100 patients are employed as a test dataset [26]. The annotations are converted from radiotherapy structure-set contours to binary label maps. A requirement prior to determining the clinimetric reliability of the developed models is verifying whether their performance is comparable to earlier published work by Neve et al. [13]. and Kujawa et al. [14], who have employed similar model architectures. For this, the following conventional geometric segmentation metrics are calculated: the Dice score, ASSD, and the 95th percentile Hausdorff distance (HD95). All metrics are evaluated with respect to each individual observer. The relative volume error (RVE) is calculated to assess the relative difference in measured tumor volume between the models and observers. In order to investigate any effect of tumor size on the model performance, model evaluations are performed on each quartile within the tumor volume distribution, as defined by the mean tumor volume of all human observers.

### Clinimetric performance evaluation: analysis of volumetric agreement limits

Clinimetric parameters are employed in order to assess the reliability of the developed models in terms of clinically relevant parameters. For this, the test results of both models are compared to the volumetric agreement limits of human observers. Observer agreement is commonly assessed using Bland-Altman plots. As a generalization of this procedure, the LOAM method is used (limits of agreement with the mean) [25], which extends the calculation to multiple observers and expresses the agreement limits as a confidence interval. Our inter-observer variability study on VS annotation in ceT1-scans is based on the LOAM, which results in a volume-dependent agreement limit [26]. The upper limit of the 95% confidence interval of the LOAM, hereafter abbreviated as the upper agreement limit, indicates the ostensible maximum deviation that a new observer would deviate from the mean of known observers. When a new observation falls outside of this upper agreement limit, the deviation cannot be fully attributed to inter-observer variability but to annotation error as well. The results of our earlier study are used to assess whether the performances of automated segmentation models fall within the limits of human inter-observer variability.

For each automatically segmented tumor volume in the test dataset, the relative deviation from the mean of human observers ($\:{{\Delta\:}}_{V\%}$) is calculated. The computed deviation is subsequently compared to the earlier calculated agreement limits (i.e. LOAM). The automated segmentation is deemed comparable to human observers if the deviation is lower than the upper agreement limit.

### Clinimetric performance evaluation: measuring inter-observer variability

To investigate how automatically obtained tumor volumes should be interpreted, our earlier study on inter-observer variability with five human observers is extended by including the results on the test dataset for both automated segmentation models individually in the overall LOAM analysis [26]. In other words, each model is added as an additional observer, allowing the calculation of new agreement limits for each model.

The confidence intervals of the LOAM analysis demonstrate the statistically probable range of the true agreement limit. Therefore, when an observer that is statistically indistinguishable from the earlier included observers is added, the confidence intervals will become more narrow. On the contrary, an additional observer that produces annotations that cannot be fully attributed to inter-observer variability, will result in novel agreement limits that are significantly different from the original limits. This postulation therefore allows for the comparison of the updated agreement limits including an AI annotator to the earlier found limits based on human observers solely. Calculations are performed using Python (Version 3.8.8) with the Matplotlib (Version 3.7.2) and NumPy (Version 1.22.4) libraries.

## Results

### Model performance

Both automated segmentation models successfully detect VSs for all 100 ceT1-scans in the test set. Table 2 presents the resulting segmentation performance metrics. For the single-center model, the median Dice score is 91.6% (IQR: 85.2–94.4), while the multi-center model shows a slightly increased performance of 91.9% (IQR: 87.3–94.7) and is comparable to other multi-centric studies (cf. 93% [13] and 91% [14]). Both the median ASSD (0.34 mm (IQR: 0.26–0.44) cf. 0.29 mm (IQR: 0.25–0.36)) and median HD95 (0.86 mm (IQR: 0.78-1.00) cf. 0.80 mm (IQR: 0.70-1.00)) are higher (i.e. worse) in the single-center model compared to its multi-center counterpart. The ASSD is similar to other studies (cf. 0.36 [13] and 0.20 [14] mm), whereas the HD95 is smaller (cf. 1.0 [13] and 1.7 [14] mm). The Dice score displays a clear dependency on tumor volume, since the performance rises with increasing tumor volume quartile.

Table 3 summarizes the resulting tumor volumes and RVE. Overall, the single-center model results in a larger median tumor volume (1,038 mm^3^ (IQR: 302-3,332)) compared to the multi-center model with 963 mm^3^ (IQR: 255-3,145). Furthermore, the multi-center model exhibits a lower median error (8.2% (IQR: 3.9–15.8)) than the single-center model: 12.4% (IQR: 6.8–27.4) and is comparable to earlier studies (cf. 7.1% [13] and 8.4% [14]). For both models, the RVE exhibits a clear volume dependency, since larger tumors have a lower volume error than small tumors. Both models display an inferior performance compared to human observers in terms of geometric segmentation metrics (see Table 2), since the median metrics for human annotators yield a Dice score of 94.1% (IQR: 91.0-96.1), ASSD of 0.20 mm (IQR: 0.15–0.27), and RVE of 4.6% (IQR: 2.1–8.7). Only a minimal difference is found for HD95, showing 0.78 mm (IQR: 0.70–0.99). A similar volume dependency for the Dice and RVE are found in human annotators. Additionally, the ASSD depends on tumor volume, since larger tumors exhibit a higher ASSD. Furthermore, in terms of tumor volume, both automated segmentation models overestimate tumor size, as human annotation results in smaller observed tumor volumes (see Table 3).

**Table 2:** Segmentation performance metrics for both models and human annotators

	Single-center model	Multi-center model	Human annotators
Dice [%]	91.6 (85.2–94.4)	91.9 (87.3–94.7)	94.1 (91.0-96.1)
Q1 Q2 Q3 Q4	80.2 (70.9–84.8) 90.0 (86.2–91.9) 93.3 (91.0-94.5) 95.5 (93.8–96.7)	82.6 (76.5–86.3) 90.7 (88.3–92.0) 93.5 (92.0-94.5) 95.9 (94.7–96.6)	88.8 (83.4–91.5) 92.9 (90.9–94.5) 95.5 (94.2–96.4) 96.5 (95.1–97.2)
ASSD [mm]	0.34 (0.26–0.44)	0.29 (0.25–0.36)	0.20 (0.15–0.27)
Q1 Q2 Q3 Q4	0.40 (0.30–0.52) 0.30 (0.24–0.40) 0.32 (0.26–0.42) 0.34 (0.27–0.44)	0.32 (0.27–0.40) 0.26 (0.22–0.33) 0.29 (0.25–0.36) 0.30 (0.26–0.36)	0.18 (0.14–0.23) 0.19 (0.15–0.26) 0.21 (0.16–0.29) 0.25 (0.18–0.32)
HD95 [mm]	0.86 (0.78-1.00)	0.80 (0.70-1.00)	0.78 (0.70–0.99)
Q1 Q2 Q3 Q4	1.00 (0.80–1.09) 0.83 (0.70-1.00) 0.83 (0.70-1.00) 0.80 (0.78-1.00)	0.82 (0.78-1.00) 0.80 (0.70-1.00) 0.80 (0.70-1.00) 0.80 (0.78-1.00)	0.78 (0.78–0.96) 0.78 (0.70-1.00) 0.78 (0.70-1.00) 0.80 (0.70–0.80)

Note. – Data is split into tumor volume quartiles and is presented as the median with inter-quartile range in parentheses

ASSD = average symmetric surface distance, HD95 = 95th percentile Hausdorff distance

**Table 3:** Volumes and relative volume error for both models and human annotators

	Single-center model	Multi-center model	Human annotators
Volume [mm^3^]	1,038 (302-3,332)	963 (255-3,145)	905 (218-3,106)
Q1 Q2 Q3 Q4	151 (95–202) 509 (354–657) 1,577 (1,341-2,396) 5,983 (4,783-8,591)	128 (88–163) 462 (328–590) 1,472 (1,269-2,299) 5,715 (4,487-8,358)	114 (73–155) 464 (321–609) 1,442 (1,198-2,245) 5,482 (4,341-8,281)
RVE [%]	12.4 (6.8–27.4)	8.2 (3.9–15.8)	4.6 (2.1–8.7)
Q1 Q2 Q3 Q4	43.4 (23.9–27.4) 15.4 (8.5–26.8) 11.6 (7.4–17.2) 5.7 (3.3–9.3)	24.5 (12.2–46.4) 8.6 (5.0-15.5) 8.4 (4.8–13.2) 3.6 (2.1-6.0)	12.5 (5.2–25.1) 5.2 (2.5-9.0) 3.4 (1.4–5.7) 2.8 (1.2–4.7)

Note. – Data is split into tumor volume quartiles and is presented as the median with inter-quartile range in parentheses.

RVE = relative volume error

**Table 4:** Number of automatic volume measurements deemed (in)comparable to human performance with corresponding deviation from the upper agreement limit

	Single-center model			Multi-center model		
	Correct	Incorrect		Correct	Incorrect	
	*N* (%)	*N* (%)	Deviation from upper agreement limit (pp)	*N* (%)	*N* (%)	Deviation from upper agreement limit (pp)
All tumors	44 (44%)	56 (56%)	7.9 (4.0-18.3)	73 (73%)	27 (27%)	4.6 (3.4–8.7)
Q1 Q2 Q3 Q4	6 (24%) 13 (52%) 7 (28%) 18 (72%)	19 (76%) 12 (48%) 18 (72%) 7 (27%)	23.3 (7.3–46.3) 10.1 (6.6–15.9) 6.3 (1.7–9.5) 2.0 (0.5–3.3)	15 (60%) 21 (84%) 13 (52%) 24 (96%)	10 (40%) 4 (16%) 12 (48%) 1 (4%)	10.7 (6.5–49.7) 2.5 (1.3–3.8) 4.4 (3.6–5.3) 0.1 (0.1–0.1)

**Note. –** Data is split into tumor volume quartiles. Deviation from upper agreement limit is presented as the median with inter-quartile range in parentheses

pp = percentage point

### Volumetric agreement limit analysis

**Fig. 2:**
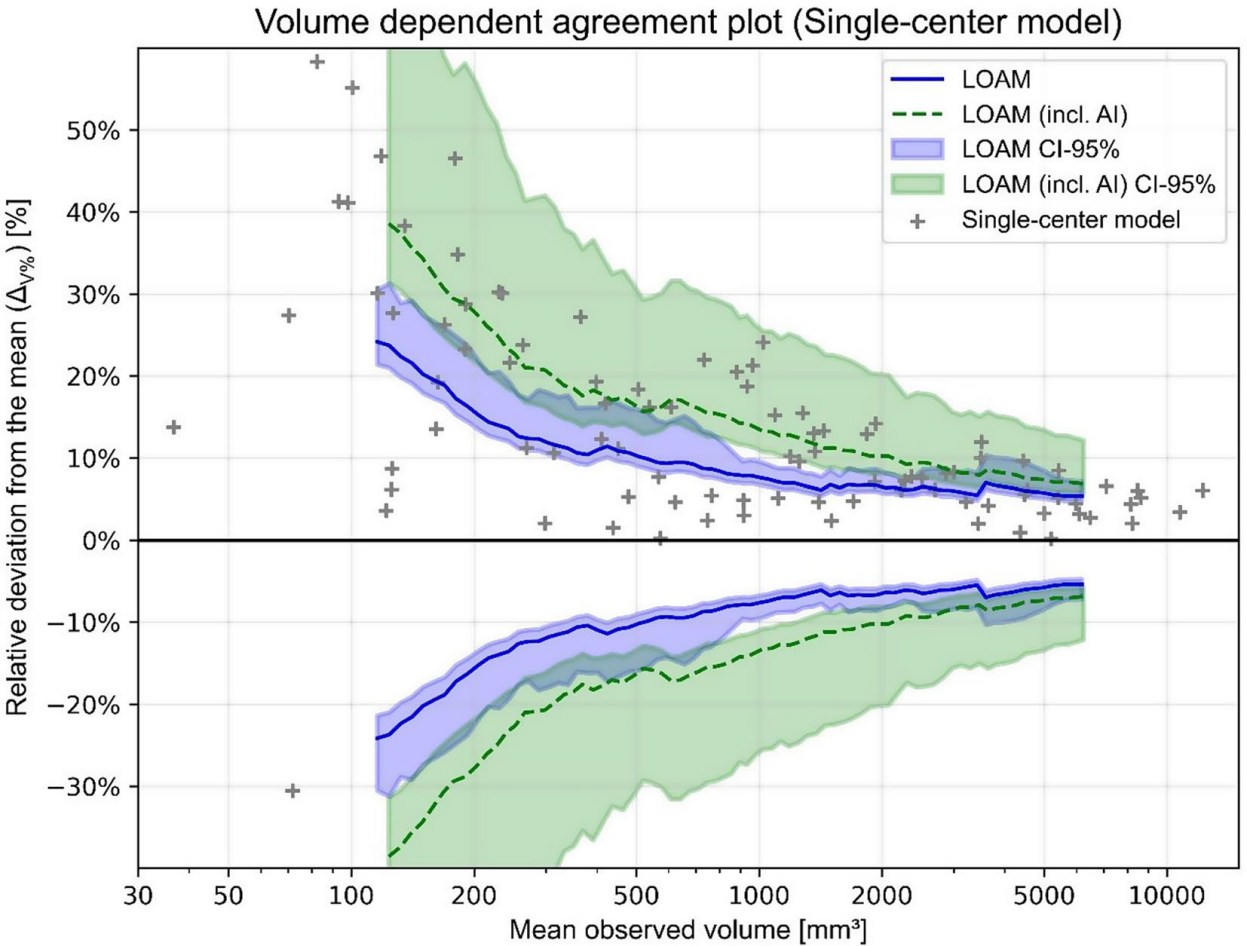
Agreement plot using a sliding window for the 100 tumors in the test set. Data points display the volumetric results of the single-center model. The previously obtained agreement limits for solely human annotators are displayed in blue, whereas green displays the inclusion of the single-center model

**Fig. 3:**
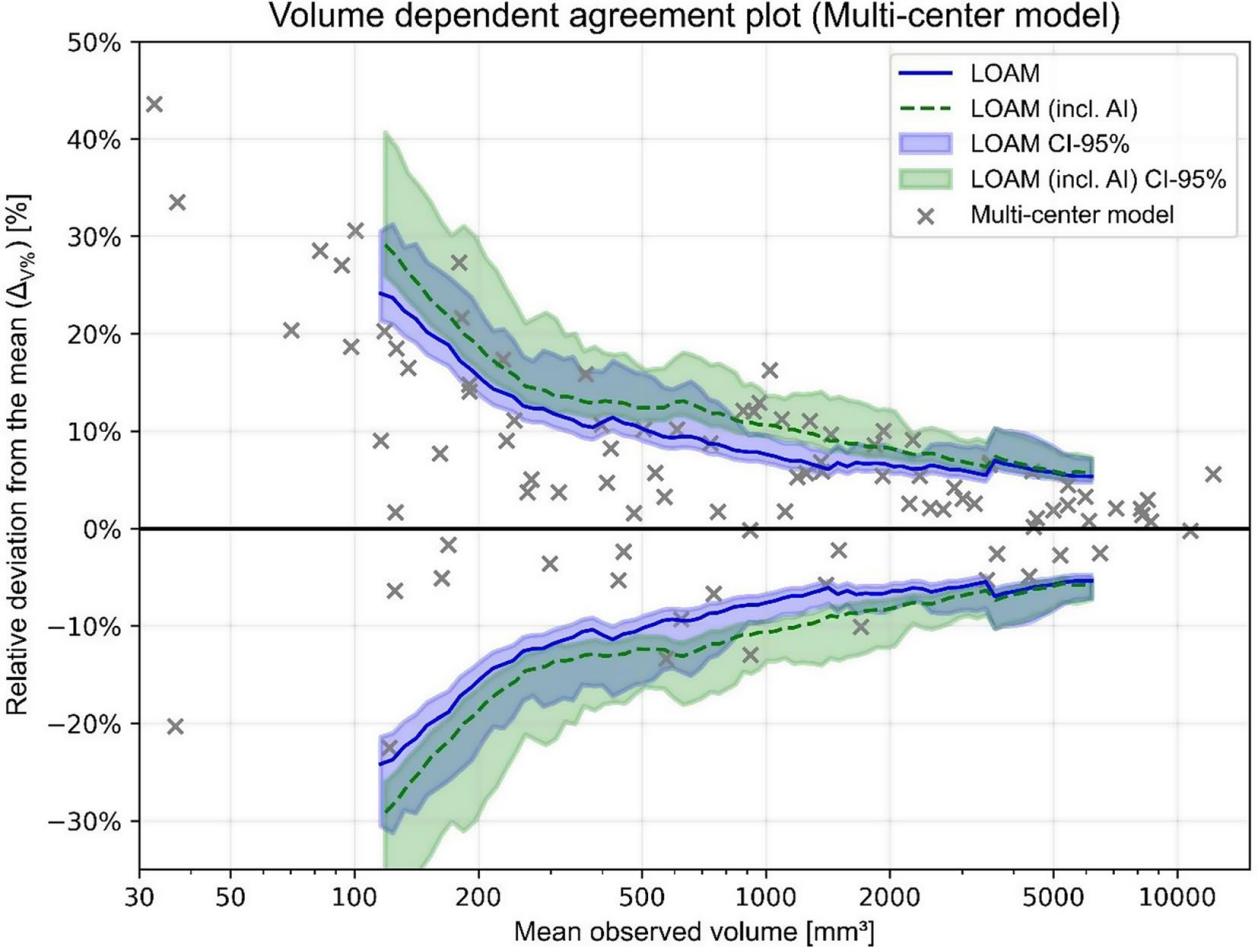
Agreement plot using a sliding window for the 100 tumors in the test set. Data points display the volumetric results of the multi-center model. The previously obtained agreement limits for solely human annotators are displayed in blue, whereas green displays the inclusion of the multi-center model

For both automated segmentation models, the relative volumetric deviation from the mean of human observers ($\:{{\Delta\:}}_{V\%}$) for each tumor is compared to the earlier calculated agreement limits (i.e. LOAM) [26]. Figures 2 and 3 show the human agreement limits with the determined $\:{{\Delta\:}}_{V\%}$ values for each individual model. An automated tumor volume is deemed comparable to human performance if $\:{{\Delta\:}}_{V\%}$ is smaller than the upper agreement limit, which is presented in Table 4.

Of the 100 tumors in the test dataset, a total of 44 tumor volumes are found comparable to human annotations for the single-center model. The remaining 56 tumors exceed the upper agreement limit by a median of 7.9% points (IQR: 4.0-18.3). The multi-center model results in 73 tumor volumes are comparable to human performance. The 27 remaining tumors display a median exceedance of 4.6% points (IQR: 3.4–8.7).

In general, automated annotated tumors are again larger than their human counterparts, since most tumor volumes have a positive relative deviation from the mean ($\:{{\Delta\:}}_{V\%}$). This tendency becomes more apparent for the single-center model, where nearly all tumor volumes display a positive $\:{{\Delta\:}}_{V\%}$.

Automated volumes of small tumors are deemed least comparable to human-annotated tumor volumes. Only 24% of the automated annotations determined by the single-center model and 60% of the multi-center model fall within human agreement limits for the first tumor volume quartile. Additionally, for volumes that exceed the upper agreement limit, a large median exceedance from the upper agreement limit is obtained for the smallest tumors: 23.3 and 10.7% points for the single and multi-center model, respectively. Alternatively, for the largest tumor volume quartile, the automated annotations are comparable to human observers (72% and 96%), while the remaining volumes only minimally exceed the upper agreement limit, being 2.0 pp and 0.1 pp for the single and multi-center model, respectively. Generally, $\:{{\Delta\:}}_{V\%}$ decreases for increasing tumor volume.

### Inter-observer variability including an AI annotator

The newly found inter-observer variability with both human and AI annotators in terms of the LOAM is displayed for both automated segmentation models in Figs. 2 and 3. In both cases, the LOAM including an AI annotator is higher and the confidence interval generally larger than for human annotators only. This indicates that the addition of automated segmentation models induces a higher inter-observer variability.

The inclusion of the single-center model causes a sharp increase in the LOAM and broadening of its confidence interval. Very little overlap remains between the two confidence intervals, which indicates a significant disparity between the two agreement limits. For higher tumor volumes, the single-center LOAM approaches the human agreement limits, though still showing a large confidence interval (Fig. 2).

When the multi-center model is included, a slight increase in the LOAM and broadening of its confidence interval can be observed. There is an overlap between the two confidence intervals on the entire tumor volume spectrum (Fig. 3).

## Discussion

Tumor monitoring is an important part of VS management, where the detection of changes in tumor size over time provides crucial information on further treatment steps. Studies have been conducted to investigate the automation of this process by exploring AI for automated volumetric tumor annotation [10–14]. These studies have yielded well-performing models in terms of commonly used segmentation performance metrics. However, these geometric-based metrics lack the ability to assess clinimetric reliability, which is a crucial step towards incorporating automated segmentation models into clinical practice. In order to investigate the clinical reliability of AI segmentation for VS patients, we need to consider the inter-observer variability. In this study, a comparison is made between automated annotations and the inherent human inter-observer variability.

Both single and multi-center segmentation models perform well in terms of the commonly evaluated geometric-based performance metrics. The Dice scores are comparable to other multi-centric studies and approach inter-human annotation similarity according to our data [13, 14]. Both models display a higher ASSD to human annotators, but are comparable to other studies [13, 14]. The HD95 is comparable across both models and human observers, though smaller than other studies [13, 14]. These results indicate that the AI models generally annotate tumors with sub-voxel accuracy compared to the ground truth, since most erroneous annotations are within single-voxel distance from the ground truth, as the median voxel spacing in the test data equals 0.78 mm. Lastly, the AI-determined tumor volumes are larger than their human counterparts for both models, which translates into a higher RVE compared to human performance. The multi-center model RVE performance is comparable to other studies [13, 14].

The presented evaluation metrics show that both single and multi-center automated segmentation models perform well and are comparable to similar models from literature. Nonetheless, the volumetric agreement limit analyses demonstrate a clear disparity between both models in terms of comparability to human performance and therewith clinimetric reliability. Only 44% of the tumor volumes estimated by the single-center model are considered comparable to human annotations, which is in contrast to the 73% for the multi-center model. This indicates that the single-center model is not suitable for volumetric evaluation in clinical practice. Regarding the inadequate annotations of the multi-center model, the median exceedance of human agreement limits is only 4.6% points. This shows that even though these annotations are statistically considered inaccurate, they are fairly close to human performance. Furthermore, the smallest tumor volume quartile contains a higher number of erroneous annotations (40%) compared to the largest quartile (1%). This corresponds to the Dice-score results, where both automated and human annotators display a higher error rate in small tumors compared to large ones. This observed volume dependency can be explained by the fact that small tumors are inherently more prone to annotation error in human annotators [26]. As automated segmentation models are trained using human annotation labels, automated segmentation of small tumors will consequently have more annotation errors as well [13]. Automatic annotation for small tumors should therefore be employed with extra vigilance.

The LOAM method has been used to estimate updated agreement limits that include each automated segmentation models as an extra observer. The inclusion of the single-center model results in poor agreement limits with an extremely broad confidence interval having limited overlap with the previously determined human-based agreement limits (see Fig. 2). This provides additional evidence that the implementation of the single-center model is infeasible for clinical practice. Contrarily, the inclusion of the multi-center model produces agreement limits that are highly similar to the human observer variability, albeit slightly larger (see Fig. 3) 

**Fig. 4:**
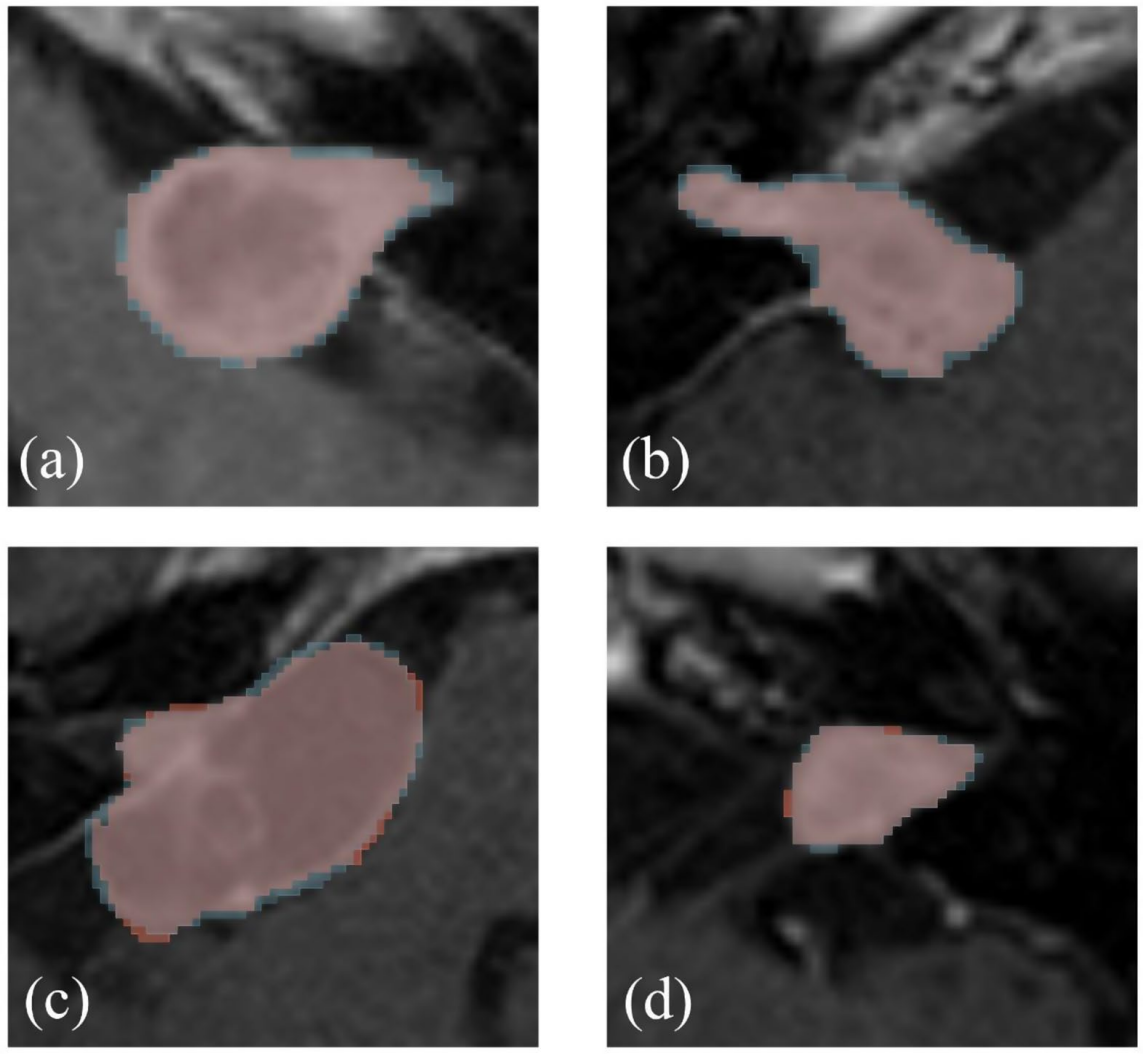
Examples of tumors in the test set with corresponding annotation of one individual human observer (red) and the single-center segmentation model (blue). All four automatic annotations resulted in similar Dice scores (range: 92.0–92.7%). Relative deviation from the mean of human observers ($\:{\varDelta\:}_{V\%}$) was found to be: (**a**) 15.9%, (**b**) 12.5%, (**c**) 0.2%, and (**d**) 2.5%

Both developed models perform comparable to other (multi-centric) studies with identical model frameworks based on the commonly used segmentation metrics [13, 14]. Additionally, no strong disparity in performance between both models is found for these metrics. However, when their clinimetric reliability is evaluated using agreement limits based on estimated tumor volume, a clear difference is observed. Herein, the multi-center model consistently outperforms the single-center model. This performance discrepancy in terms of metric types demonstrates that satisfactory segmentation metrics do not necessarily imply a well-performing and reliable model for use in clinical practice. Figure 4 displays this disparity by showing four tumors with a similar, high Dice score, although with a clearly varying relative deviation from the mean of human observers ($\:{{\Delta\:}}_{V\%}$). Consequently, even though the single-center model displays adequate scores for the segmentation metrics, the model is considered too unreliable and imprecise for clinical practice. Conversely, the results from the multi-center model demonstrate that this model performs nearly as well as human annotators in terms of clinimetrics. The difficulty of translating geometric-based segmentation metrics, especially the Dice score, to clinically relevant interpretations has been observed in earlier studies [17, 19, 21–23]. Therefore, in order to thoroughly evaluate model performance and reliability in practice, future research in automated tumor segmentation for volumetry should not only report segmentation metrics, but incorporate clinimetrics that are relevant to the domain of clinical application as well.

The applicability of our findings is limited by several factors. Firstly, the sole use of ceT1-weighted images in training both AI models limits the application in clinical practice, since a considerable proportion of medical centers follow-up VS patients using exclusively T2-weighted images. The reason for this is to reduce costs and the potential adverse effects of contrast agents in patients [1, 11, 32]. However, studies have shown that AI can be an effective tool for automated tumor segmentation on T2-images as well [10, 11, 13, 14]. Nevertheless, similar to this study, their performance should be evaluated using clinically relevant metrics, in order to truly assess their clinimetric reliability and therewith their usability in daily clinical practice. Secondly, our developed models are not externally validated. MR imaging is intrinsically highly heterogeneous, due to its qualitative nature and the large number of different imaging protocols [14]. This all encumbers the generalization capabilities of most deep learning models and thereby possibly the applicability of our findings to other centers. The developed multi-center model can therefore only reliably be used in-house. Lastly, caution is warranted when employing the model for small tumors, as the model is less accurate for small tumor volumes. This limitation can be attributed to both the undersampling of small tumors in the training dataset, and the inherent higher annotation error rate in the human annotated ground truth labels for small tumors [26]. To advance our findings to full multi-center clinical application and to alleviate the limitations, a broad multi-center model in terms of both imaging parameters and tumor volumes should be developed that is externally validated using clinimetrics.

Overall, both developed models perform well in terms of the commonly used segmentation metrics. However, when compared to human-based agreement limits, only the multi-center model demonstrates a clinimetric reliability that would justify its use in clinical practice. Hence, satisfactory geometric-based segmentation metrics do not necessarily imply reliable models for clinical implementation. Updated agreement limits, with the addition of the multi-center model as an observer, are slightly higher than for solely human observers. This further exemplifies the clinimetric reliability of the multi-center automated segmentation model and thereby a feasible employment of such model in clinical practice. However, due to the volume-dependent model performance, its employment for small tumors should be done with caution.

## Data Availability

Data that support the findings of this study are available from the corresponding author upon reasonable request.
